# Activated c-Kit receptor in the heart promotes cardiac repair and regeneration after injury

**DOI:** 10.1038/cddis.2016.205

**Published:** 2016-07-28

**Authors:** S Di Siena, R Gimmelli, S L Nori, F Barbagallo, F Campolo, S Dolci, P Rossi, M A Venneri, E Giannetta, D Gianfrilli, L Feigenbaum, A Lenzi, F Naro, E Cianflone, T Mancuso, D Torella, A M Isidori, M Pellegrini

**Affiliations:** 1 Department of Anatomical, Histological, Forensic and Orthopedic Sciences, Sapienza University, Rome, Italy; 2 Department of Experimental Medicine, Sapienza University, Rome, Italy; 3 Department of Medicine and Surgery, University of Salerno, Baronissi, Italy; 4 Department of Biomedicine and Prevention, Tor Vergata University, Rome, Italy; 5 Leidos Biomedical Research, Inc., Frederick National Laboratory for Cancer research, Frederick, MD, USA; 6 Department of Medical and Surgical Sciences, Magna Graecia University, Catanzaro, Italy; 7 Institute of Cell Biology and Neurobiology, CNR, Rome, Italy

## Abstract

The role of endogenous c-Kit receptor activation on cardiac cell homeostasis and repair remains largely unexplored. Transgenic mice carrying an activating point mutation (TgD814Y) in the kinase domain of the *c-Kit* gene were generated. c-Kit^TgD814Y^ receptor was expressed in the heart during embryonic development and postnatal life, in a similar timing and expression pattern to that of the endogenous gene, but not in the hematopoietic compartment allowing the study of a cardiac-specific phenotype. *c-Kit*^*TgD814Y*^ mutation produced a constitutive active c-Kit receptor in cardiac tissue and cells from transgenic mice as demonstrated by the increased phosphorylation of ERK1/2 and AKT, which are the main downstream molecular effectors of c-Kit receptor signaling. In adult transgenic hearts, cardiac morphology, size and total c-Kit^+^ cardiac cell number was not different compared with wt mice. However, when *c-Kit*^*TgD814Y*^ mice were subjected to transmural necrotic heart damage by cryoinjury (CI), all transgenic survived, compared with half of wt mice. In the sub-acute phase after CI, transgenic and wt mice showed similar heart damage. However, 9 days after CI, transgenic mice exhibited an increased number of c-Kit^+^CD31^+^ endothelial progenitor cells surrounding the necrotic area. At later follow-up, a consistent reduction of fibrotic area, increased capillary density and increased cardiomyocyte replenishment rate (as established by BrdU incorporation) were observed in transgenic compared with wt mice. Consistently, CD45^−^c-Kit^+^ cardiac stem cells isolated from transgenic *c-Kit*^*TgD814Y*^ mice showed an enhanced endothelial and cardiomyocyte differentiation potential compared with cells isolated from the wt. Constitutive activation of c-Kit receptor in mice is associated with an increased cardiac myogenic and vasculogenic reparative potential after injury, with a significant improvement of survival.

c-Kit is a tyrosine kinase receptor essential for proliferation, survival and migration of several stem cell types such as melanocyte precursors, hematopoietic and germ stem cells.^1, 2, 3, 4^ More recently, c-Kit receptor was reported to be expressed in cardiac and neuronal stem cells.^5, 6^ Mice lacking *c-Kit* gene present germ cell and melanocyte defects and die in the first days of postnatal life because of impaired hematopoiesis.^7, 8^ The binding of c-Kit ligand (KL) induces receptor homodimerization and autophosphorylation of the intracellular tyrosine kinase domains leading to the modulation of different signaling pathways such as AKT and MAPKs.^9, 10, 11^

In the past 15 years, several studies have shown that c-Kit^+^ cardiac stem cells (CSCs) have beneficial effects in cardiac repair and regeneration.^12^ Genetically mutant mice deficient in c-Kit signaling (*W/Wv*) show a worsened cardiac remodeling after myocardial infarction, conversely transgenic mice overexpressing KL in the heart exhibit an improved myocardial repair compared with their wt littermates after myocardial infarction.^13, 14, 15^ Increased expression of KL occurring in ischemic heart favors migration of c-Kit^+^ bone marrow-derived and CSCs via activation of p38 MAPK.^16, 17, 18^ Furthermore, c-Kit^+^ CSCs have been recently used in the treatment of patients with ischemic cardiomyopathy and heart failure with significant improvement in systolic function and a reduction in infarct size.^19, 20, 21^

Although c-Kit is widely used as a 'cell surface marker' to identify stem cells from the embryonic as well as adult heart,^22, 23^ little information^14, 15^ is available on the function that c-Kit signaling has in the activation of CSCs in cardiac repair.

To investigate the role of c-Kit in heart repair, transgenic mice were generated carrying a mutated version of *c-Kit* gene. The substitution of tyrosine for aspartic acid 814 in the phosphotransferase domain leads to constitutive activation of the receptor. Decreased fibrotic area in cryoinjured hearts, reduced inflammatory myeloid cells in the blood, increased number of c-Kit^+^CD31^+^ endothelial cells and isolectin B4 (IB-4)-labeled capillaries as well as BrdU-positive newly formed cardiomyocytes in damaged cardiac area of transgenic mice were observed. MAPK and AKT activation was significantly enhanced in the hearts and CSCs of transgenic mice, whereby the two kinases modulate the activation and endothelial/myogenic differentiation of CSCs. Overall, these data indicate that the activated c-Kit receptor exerts a beneficial protective/regenerative role for myocardial tissue after injury improving cardiac remodeling and repair while fostering differentiation of cardiac progenitor cells likely due to MAPK and AKT signaling activation.

## Results

### Generation of transgenic mice expressing an activated c-Kit receptor in heart

**Figure 1:**
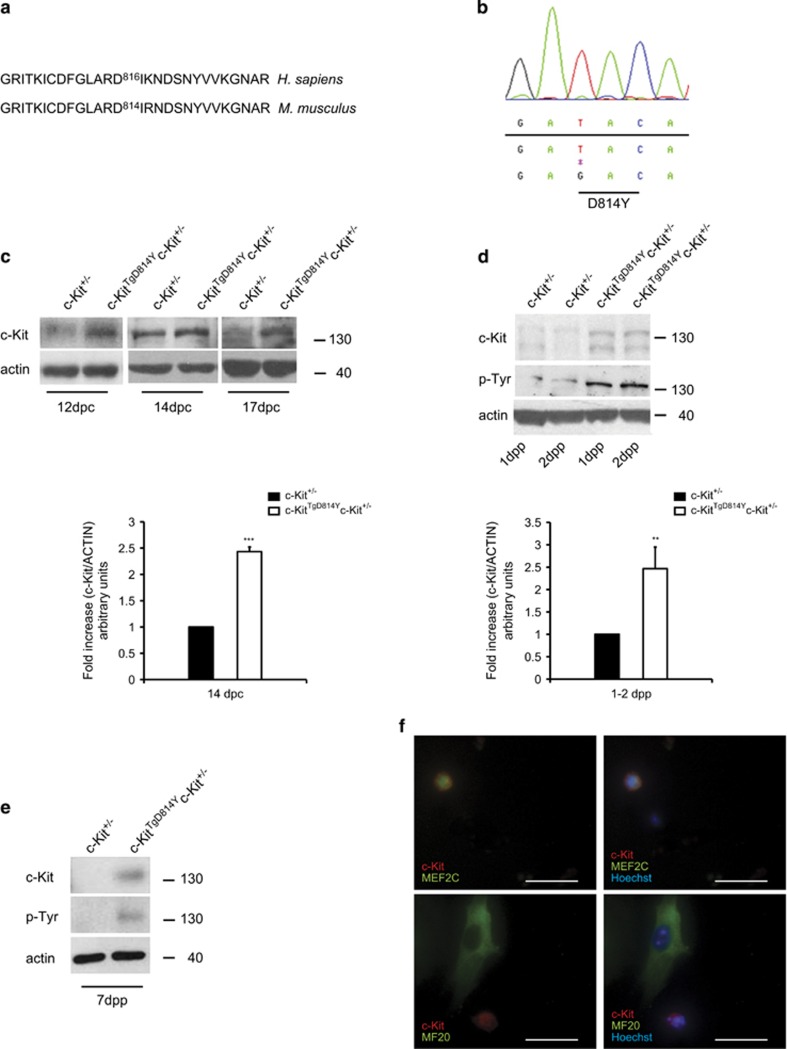
D814Y substitution induces a constitutive c-Kit activation. (**a**) Pairwise local alignments of human (*Homo sapiens*) and mouse (*Mus musculus*) c-Kit protein sequences surrounding the putative residue for constitutive activation in the second kinase domain of the receptor. (**b**) Sequence chromatogram showing the aspartic acid to tyrosine mutation introduced in *c-Kit* mouse BAC RP23- 309C11. (**c**) WB and densitometry of c-Kit expression on hearts collected from embryos at different stages of development. Mutant c-Kit protein is expressed 2.5-fold higher than endogenous level in *c-Kit*^*TgD814Y*^*c-Kit*^*+/−*^ embryos. (**d**) WB and densitometry of c-Kit expression on total heart lysate collected from neonatal mice. Mutant c-Kit protein is expressed 2.5-fold higher than endogenous c-Kit protein levels in *c-Kit*^*TgD814Y*^*c-Kit*^*+/−*^ mice. c-Kit activation is shown by a pan phospho-tyrosine antibody that recognizes a specific band at the receptor height. (**e**) WB analysis on hearts collected from 7 dpp mice. Data are obtained from at least three separate hearts and reported±S.D.; **P<0.01, ***P<0.001. (**f**) Immunofluorescence analysis of c-Kit^+^ cells (red) in isolated *c-Kit*^*TgD814Y*^ neonatal myocytes cultured for 24 h co-stained with MEF2C (green) and MF20 (green). Nuclei (blue) were counterstained with Hoechst 33342. Scale bars 30 *μ*m

To generate transgenic mice expressing a constitutively activated c-Kit receptor, a bacterial artificial chromosome (BAC) reconstitution method was used allowing the transcription of *c-Kit* gene by endogenous regulatory sequences (Figure 1).

In humans, the 816 aspartic amino acid of the second kinase domain of c-Kit was found to be substituted with activating mutations in several tumors (Figure 1a).^24, 25, 26^ The corresponding murine 814 aspartic residue was therefore successfully mutated to tyrosine (D814Y) in the mouse BAC (RP23-309C11) as confirmed by sequence analysis (Figures 1a). The resulting BAC was subsequently used to generate transgenic mice. Several founder lines were produced, two of which (Tg7 and Tg8) transmitted and similarly expressed the constitutive active c-Kit receptor (c-Kit^TgD814Y^).

**Figure 2:**
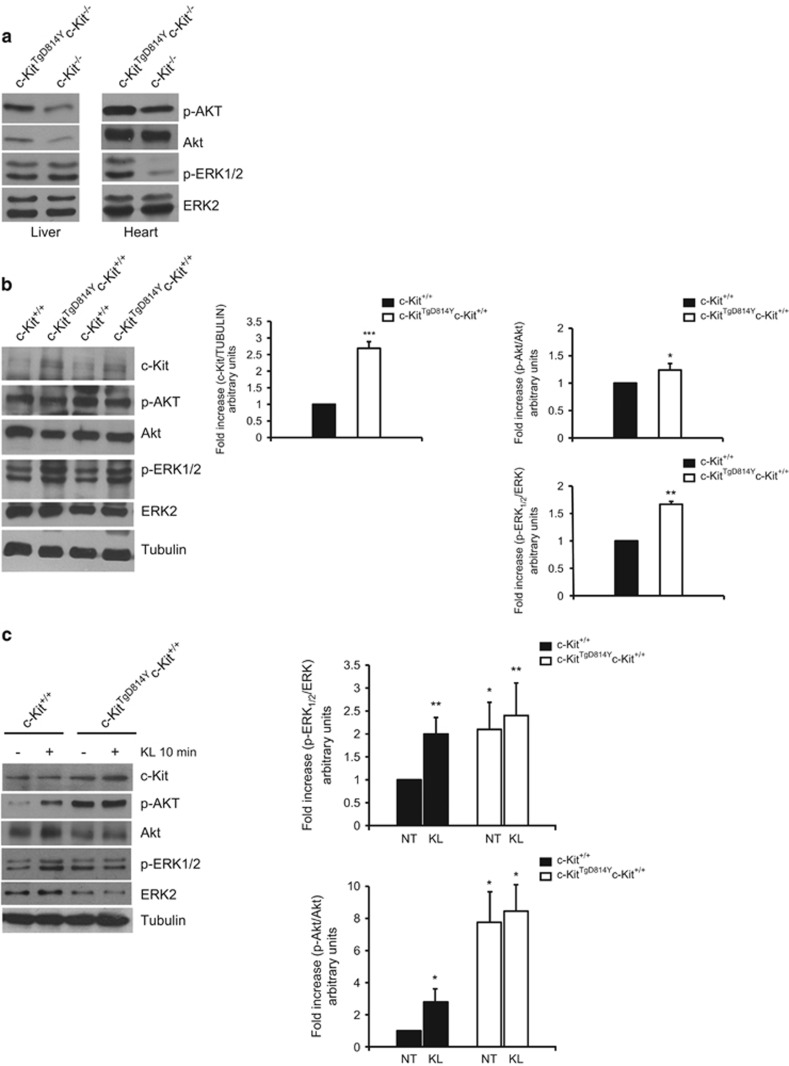
MAPK and AKT activation in *c-Kit*^*TgD814Y*^ transgenic hearts. (**a**) Hearts and livers from *c-Kit*^*TgD814Y*^*c-Kit*^*−/−*^ and *c-Kit*^*−/−*^ E15.5 were isolated and protein extracts analyzed for AKT and MAPK phosphorylation. Phosphorylated substrates were increased in embryonic hearts from transgenic mice. (**b**) WB analysis on protein extracts from *c-Kit*^*TgD814Y*^*c-Kit*^*+/+*^ and *c-Kit*^+/+^ 3 dpp hearts of two different animals. Densitometry of c-Kit expression, MAPK and AKT phosphorylation is reported. Data are reported as mean of three hearts±S.D.; *P<0.05, **P<0.01, ***P<0.001. (**c**) Neonatal cardiac cells isolated from *c-Kit*^*TgD814Y*^*c-Kit*^*+/+*^ and *c-Kit*^+/+^ 3 dpp hearts were stimulated with 100 ng/ml of KL for 10 min and protein extracts processed for AKT and MAPK phosphorylation. Densitometry of six separate experiments is reported. Data are reported±S.D.; *P<0.05, **P<0.01

Transgenic mice were crossed with *c-Kit* wild-type (*c-Kit*^*+/+*^) or *c-Kit* heterozygous (*c-Kit*^*+/*^^*−*^) mice. In embryonal (Figure 1c) and neonatal (Figure 1d and Figure 2b) hearts of transgenic mice expressed the receptor 2.5-fold higher when compared with control mice, indicating that c-Kit^TgD814Y^ protein expression was similar to receptor endogenous levels of *c-Kit*^+/+^ mice. At 1 week of age, c-Kit receptor could be readily detected by western blotting (WB) analysis on total heart lysates from *c-Kit*^*TgD814Y*^*c-Kit*^*+/−*^ mice, but not from *c-Kit*^*+/−*^ mice in which it was detected only by immunoprecipitation (Figure 1e,Supplementary Figure 1A). To verify whether the introduction of the D814Y mutation induced the activation of c-Kit receptor, a pan antibody against phospho-tyrosine was used in WB analyses, allowing the detection of all putative receptor autophosphorylation sites. Figures 1d and e show an increased tyrosine phosphorylation in a protein band corresponding to c-Kit receptor in transgenic hearts compared with heterozygous hearts. These results were confirmed in hearts of *c-Kit*^*TgD814Y*^*c-Kit*^*+/+*^mice (Figure 2c and data not shown). To further characterize the transgene expression, isolated neonatal cardiac cells from *c-Kit*^*TgD814Y*^*c-Kit*^*+/−*^ mice were obtained and cultured for 24 h before immunofluorescence staining. Figure 1f shows that c-Kit is expressed in myocyte progenitors as revealed by the co-staining with MEF2C marker (Figure 1f, upper panels) but not in terminally differentiated myocytes as shown by MF20 staining (Figure 1f, lower panels). These results show that the expression of the constitutively activated receptor did not prevent normal myocyte differentiation and did not induce ectopic expression of c-Kit in differentiated myocytes. Transgenic protein expression and activation were also observed in testis and cerebellum at different ages (Supplementary Figure 1B). These data confirm that transcription of *c-Kit* gene, with BAC reconstitution method, occurred under endogenous regulatory mechanisms in these cell types.

### Activated c-Kit^TgD814Y^ receptor is not expressed in hematopoietic compartments

*c-Kit* knockout mice die after birth because of severe impairment of hematopoiesis. The mutated and constitutively active c-Kit receptor protein was not detected in adult bone marrow by WB and flow cytometry analyses (Supplementary Figures 1C and D). This might reflect the reported absence of expression of the BAC construct within the bone marrow hematopoietic cells.^27^

In order to extensively study the expression of *c-Kit*^*TgD814Y*^ transgene in hematopoietic compartments, we generated *c-Kit*-deficient mice *(c-Kit*^*−/−*^; Supplementary Figure 1E) and we reconstituted them with the transgene (*c-Kit*^*TgD814Y*^*c-Kit*^*−/−*^). Similarly to *c-Kit*^*−/−*^ embryos and newborns, *c-Kit*^*TgD814Y*^*c-Kit*^*−/−*^ pups were extremely anemic, smaller than their littermates and all died before birth (Supplementary Figure 1E), indicating that the transgene does not rescue the lethal phenotype of *c-Kit* knockout mice. The lethality of *c-Kit*^*TgD814Y*^*c-Kit*^*−/−*^ mice was caused by the lack of transgene expression in fetal liver. A marginal c-Kit expression, insufficient to rescue the phenotype, was observed in both *c-Kit*^*−/−*^ and *c-Kit*^*TgD814Y*^*c-Kit*^*−/−*^ livers from E15.5 probably owing to the strategy used for *c-Kit*^*−/−*^ mouse generation (Supplementary Figures 1F and G and methods section). On the contrary, immunofluorescence analyses revealed c-Kit transgene expression in embryonic *c-Kit*^*TgD814Y*^*c-Kit*^*−/−*^ cardiac tissue, whereas, as expected, no expression was observed in any of the *c-Kit*^*−/−*^ embryos (Supplementary Figure 1F). Transgene c-Kit expression in the heart other than in head, branchial arch and stomach was also observed at earlier time of development (Supplementary Figure 1G). These observations extend the knowledge of c-Kit expression to early heart development ^28, 29^ and also point out the important notion that c-Kit^TgD814Y^transgene is expressed in the heart but not in fetal and adult hemopoietic tissues. Thus, this transgenic mouse model offers a unique advantage over the previous mouse models, as it does exclude that any cardiac phenotype is secondary to activated c-Kit receptor within bone marrow cells.

### Activated c-Kit receptor induces ERK1/2 and AKT phosphorylation

c-Kit receptor downstream signaling mainly involves MAPK and PI3K pathways.^10, 11, 30^ In order to evaluate whether these pathways were activated in *c-Kit*^*TgD814Y*^ mice, E15.5 *c-Kit*^*TgD814Y*^*c-Kit*^*−/−*^ and *c-Kit*^*−/−*^ whole liver and heart protein extracts were assessed for ERK1/2 and AKT phosphorylation (Figure 2a). No different phosphorylation levels of ERK1/2 and AKT were observed in liver extracts from *c-Kit*^*TgD814Y*^*c-Kit*^*−/−*^ compared with knockout mice (Figure 2a) as expected from the absence of the transgene expression in the liver (Supplementary Figure 1F). Conversely, in cardiac samples of *c-Kit*^*TgD814Y*^*c-Kit*^*−/−*^ mice, a small but significant increase in AKT phosphorylation and a strong increase in ERK1/2 phosphorylation were observed compared with *c-Kit*^*−/−*^ mice (Figure 2a). Similar increases of ERK1/2 and AKT phosphorylation were observed in total heart lysates from 3 dpp *c-Kit*^*TgD814Y*^*c-Kit*^*+/+*^ mice compared with *c-Kit*^*+/+*^ mice (Figure 2b). In addition, the higher phosphorylation of ERK1/2 and AKT was also observed in transgenic *c-Kit*^*TgD814Y*^*c-Kit*^*+/+*^ compared with *c-Kit*^*+/+*^ freshly isolated neonatal cardiac cell preparations (Figure 2c). Interestingly, the activation of c-Kit by its endogenous ligand (KL) induces ERK1/2 and AKT phosphorylation in cells isolated from wt hearts up to the level detected in cells from the transgene. Moreover, KL was not able to increase any further the ERK1/2 and AKT phosphorylation level in cardiac c-Kit^+^ cells isolated from transgenic animals (Figure 2c). These results demonstrate that MAPK and PI3K pathways are activated in hearts of *c-Kit*^*TgD814Y*^ mice mimicking endogenous ligand stimulation. Pre-treatment with Imatinib was able to prevent KL stimulation of wt cells, confirming the selectivity of c-Kit/KL system activation (Supplementary Figure 2A). As expected, Imatinib did not affect c-Kit^TgD814Y^ cells, because of the conformational changes induced by the mutation that modify the catalytic domain (Supplementary Figure 2A).

Finally, as a proof of principle of the constitutive activation of c-Kit^TgD814Y^ receptor, transgenic mice expressing a wt version of c-Kit receptor (c-Kit^TgWT^) from the original BAC RP23-309C11 were crossed to C57/BL6 mice for evaluation of c-Kit expression and activation. As shown in Supplementary Figure 2B, hearts from *c-Kit*^*TgWT*^ mice expressed twice the amount of receptor compared with control littermates, but no difference in autophosphorylation or activated ERK1/2 and AKT signaling pathways were detected by WB analyses.

### Activated c-Kit receptor expression does not affect cardiac development and morphology

To evaluate the effects of c-Kit receptor activation on cardiac tissue and structure, we first established whether c-Kit constitutive activation affected the number of total cardiac c-Kit^+^ cells in *c-Kit*^*TgD814Y*^ mutant mice. To simplify the read-out of quantitative immunohistochemistry and FACS analysis, we crossed *c-Kit*^*TgD814Y*^ mutant mice with transgenic mice expressing EGFP under the regulatory sequences of c-Kit promoter (*pKit*^*EGFP*^). ^31, 32^

**Figure 3:**
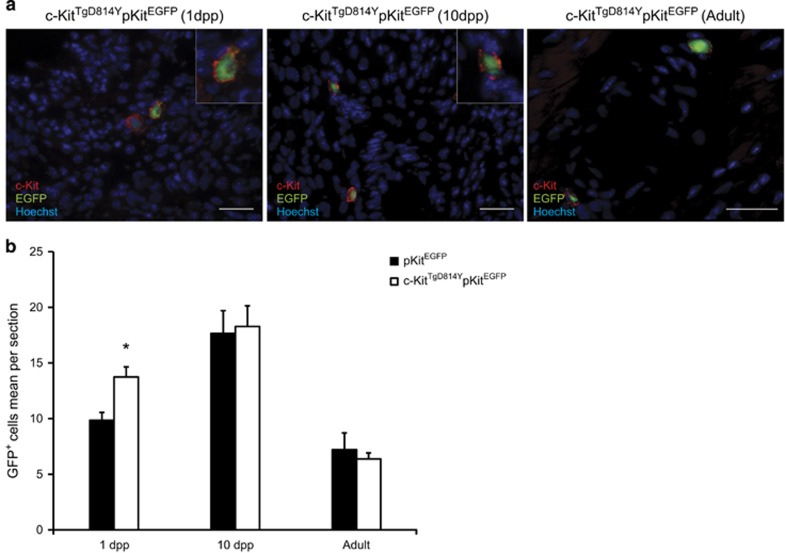
c-Kit^+^ cardiac cell distribution is not affected by *c-Kit*^*TgD814Y*^ mutation *in vivo*. (**a**) Immunofluorescence on heart sections prepared from 1 dpp, 10  dpp and adult *c-Kit*^*TgD814Y*^*pKit*^*EGFP*^ mice showing intramyocardial (1 dpp and Adult) and peri-vasal (10 dpp) c-Kit^+^ cells localization. Insets show c-Kit (red) and EGFP (green) co-expression. Scale bars 30 *μ*M. (**b**) Count of EGFP^+^ cells in *c-Kit*^*TgD814Y*^*pKit*^*EGFP*^ *versus pKit*^*EGFP*^mice at 1 dpp, 10 dpp and Adult. At least 20 sections were analyzed for each heart (*n*=3 mice for each age and genotype). The entire heart was sectioned and analyzed for 1 dpp mice. Data are reported±S.E.; *P<0.05

In *c-Kit*^*TgD814Y*^*pKit*^*EGFP*^ double transgenic hearts, the majority (over 95%) of c-Kit-expressing cells identified by the staining with an anti-c-Kit antibody co-expressed EGFP protein (Figure 3a). Histological analysis of cardiac section from mice at 1 dpp, 10 dpp and 2 months showed that c-Kit^+^EGFP^+^ cells were distributed along the epicardium, spread into the myocardium and less frequently clustered in the endocardium. Importantly, the number of EGFP^+^ cells was comparable in *c-Kit*^*TgD814Y*^*pKit*^*EGFP*^ hearts and *pKit*^*EGFP*^ at all time points (Figure 3b). Furthermore, myocardial capillary density and myocyte sizes were similar among *c-Kit*^*TgD814Y*^*pKit*^*EGFP*^ and *pKit*^*EGFP*^ hearts (data not shown). Thus, these data show that constitutive activated c-Kit receptor does not change total c-Kit^+^ cell number, myocardial vascularization and myocyte growth during development and adult cell homeostasis, at least at the age we investigated.

### Activated c-Kit receptor expression improves cardiac repair after injury

**Figure 4:**
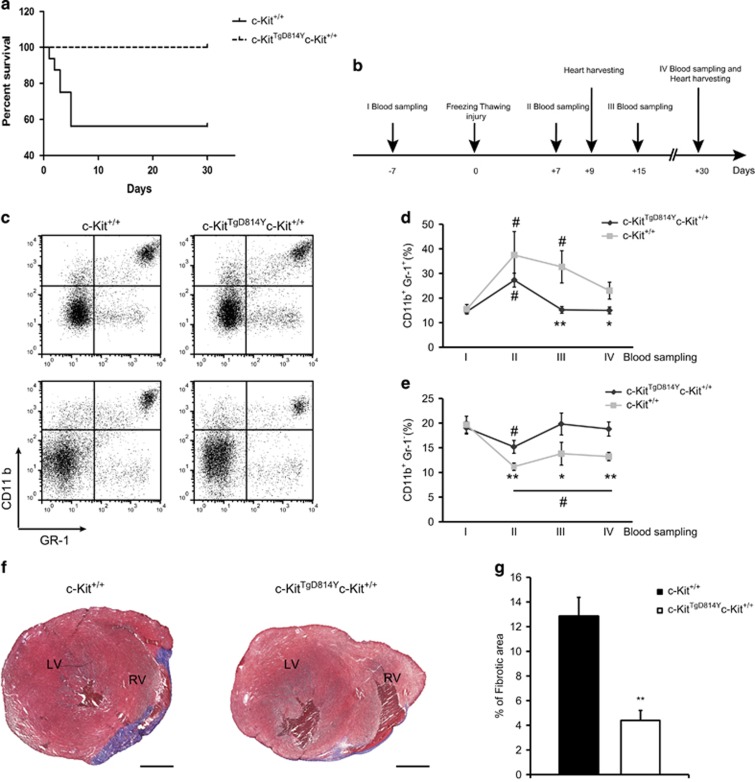
Increased survival and reduced cardiac fibrosis in *c-Kit*^*TgD814Y*^ mutants after CI. (**a**) Kaplan–Meier survival analysis in *c-Kit*^*+/+*^ (*n*=20) and *c-Kit*^*TgD814Y*^ (*n*=12) mice. Survival of *c-Kit*^*TgD814Y*^ mice was statistically significant compared with *c-Kit*^*+/+*^ 5 days after injury (100% *c-Kit*^*TgD814Y*^ *versus* 57% wt). Mice that died during surgery were not considered for the survival curve. (**b**) Timeline showing blood samplings before and after CI and hearts collection. Myocardial regeneration was assessed at 1, 4 and 6 weeks post CI. (**c**–**e**) Cytofluorimetric analyses on blood samples harvested 1 week before CI and every week until the mice were killed stained for GR-1 and CD11b surface markers. Representative plotting of GR-1 and CD11b staining of blood harvested from *c-Kit*^*+/+*^ and *c-Kit*^*TgD814Y*^ mice 7 days before (upper panels) and 30 days after (lower panels) CI. (**c**) Curves representing the mean values of CD11b^+^GR-1^+^ (**d**) and CD11b^+^GR-1^−^ (**e**) blood cells (*n*=8 mice for each group). Data are reported±S.E. Significance respect to the first blood sampling either for *c-Kit*^*+/+*^and *c-Kit*^*TgD814Y*^ is reported, ^#^*P*<0.05. Significance between *c-Kit*^*+/+*^and *c-Kit*^*TgD814Y*^ blood samples is reported at different time points, *P<0.05 **P<0.01. (**f**) Representative Masson Trichromic-stained myocardial sections from wt and transgenic mice 1 month post-myocardial lesion. Blue, fibrotic tissue and red, viable myocardium. Scale bars 1mm. (**g**) Histogram showing the percentage of fibrotic area in wt and transgenic mice (*n*=7 and *n*=9 for *c-Kit*^*+/+*^ and *c-Kit*^*TgD814Y*^ mice, respectively) 1 month post CI. Areas are measured using ImageJ software. Data are reported±S.E. **P<0.01

To challenge the role of activated c-Kit cells in myocardial repair, ventricular damage was induced by cryoinjury (CI) in hearts of 2-month-old transgenic and wt mice (Figure 4). Animal survival, systemic inflammation and cardiac tissue remodeling were evaluated to assess the recovery after cardiac injury.

As shown in Figure 4a, 43% of *c-Kit*^*+/+*^ mice died within 5 days after the injury, whereas *c-Kit*^*TgD814Y*^*c-Kit*^*+/+*^ mice were resistant to the insult as no mice died after surgery. This result indicates that transgenic mice were protected from the acute damage. Blood samples were collected 1 week before heart injury and 1, 2 and 4 weeks after the damage to monitor the level of circulating inflammatory cells. The relative amounts of CD11b^+^Gr1^+^ and CD11b^+^Gr1^−^ myeloid cells were analyzed by flow cytometry (Figures 4b–e). These two myeloid cell subsets have been considered as indicators of tissues inflammation and remodeling, respectively.^33, 34^ Figures 4c–e show pro-inflammatory CD11b^+^Gr1^+^ cells increasing in both wt and transgenic mice 1 week after damage. After 2 weeks, the percentage of CD11b^+^Gr1^+^ cells decreased to the baseline value detected before the damage in transgenic mice, whereas their percentage remained high in wt mice (Figures 4c and d). CD11b^+^Gr1^−^ cell percentage dropped immediately after CI in both treated type of animals but only in transgenic mice returned to the basal level within 2 weeks (Figures 4c and e). These data demonstrate that CD11b^+^Gr1^+^ and CD11b^+^Gr1^−^ cells are differentially modulated in transgenic mice compared with wt animals after heart injury. These data also suggest that the relative percentage of circulating CD11b^+^Gr1^+^ and CD11b^+^Gr1^−^ in the blood parallel the increased survival of transgenic mice compared with wt.

To directly assess the damage determined by CI, the extent of fibrotic area was measured by Masson Trichromic staining 3, 9 and 30 days after damage (Figures 4f and g and Supplementary Figures 3A and B). While 3 days after damage, no fibrotic tissue was observed (data not shown), 9 days after damage a large Masson Trichromic-stained myocardial area was found in both wt and transgenic mice (Supplementary Figures 3A and B). At this time, a similar level of ventricular cell wall apoptosis was found in both wt and transgenic mice (Supplementary Figure 3C). However, 30 days after damage, the fibrotic area was threefold smaller in transgenic mice compared with wt animals (12.83±4% *versus* 4.39%±2.44%) (Figures 4f and g and Supplementary Figure 3D). Concurrently, at 30 days after damage, cardiomyocyte size in the border zone of transgenic mice were smaller when compared with wild-type mice (Supplementary Figure 3E).

These results suggest that mice carrying a constitutively activated c-Kit receptor have an advantage both in the acute response to myocardial injury as shown by an increased survival, as well as in the long term as shown by a more efficient repair of necrotic tissue.

### Activated c-Kit receptor expression enhances endothelial and cardiomyocyte regeneration after injury *in vivo*

**Figure 5:**
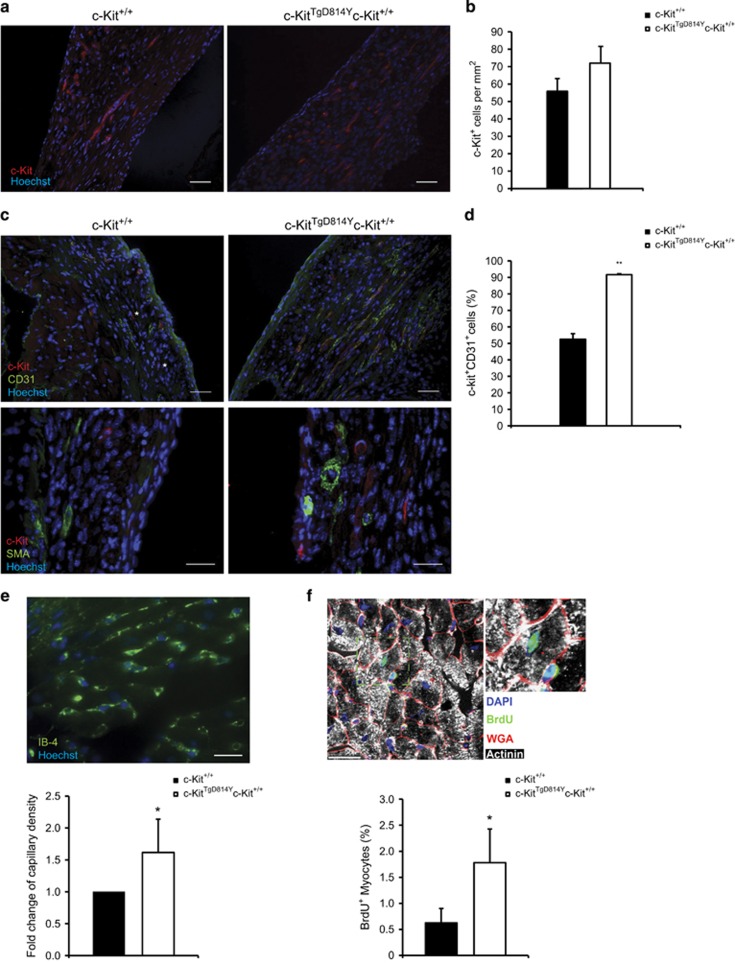
c-Kit activation induces endothelial and cardiac differentiation in *c-Kit*^*TgD814Y*^ hearts. (**a**) Immunofluorescence of c-Kit (red) 9 days after injury in *c-Kit*^*TgD814Y*^ and *c-Kit*^*+/+*^ heart sections. Scale bar 30 *μ*m. (**b**) Histogram of c-Kit^+^ cells (*n*=3 sections for 3 different mice were analyzed). Data are reported±S.E. (**c**) Immunofluorescence for c-Kit (red), CD31, smooth muscle actin (green) in *c-Kit*^*TgD814Y*^ and *c-Kit*^*+/+*^ heart sections of 9 days after CI. Asterisk indicates c-Kit single positive cells. Scale bars 30 *μ*m. (**d**) Histogram of c-Kit^+^ and CD31^+^ cells within the damaged area (*n*=3 sections for 3 different mice are analyzed). Data are reported±S.E. **P<0.01. (**e**) Representative image of *c-Kit*^*TgD814Y*^ right ventricle 30 days after CI, stained with IB-4 (green). Histogram bar graph represents the fold change of IB-4^+^ cells found in the peri-damaged area (*n*=2 sections for 3 different mice were analyzed). Scale bar 20 *μ*m. Data are reported ±S.D. *P<0.05. (**f**) Representative image of *c-Kit*^*TgD814Y*^ right ventricle 30 days after heart CI and BrdU incorporation. Inset shows *α*-actinin^+^/BrdU^+^/WGA^+^ mono-nucleated cardiomyocyte. Scale bar 50 *μ*m. Percentage of BrdU incorporating cardiomyocytes are reported in the histogram (*n*=3 sections for 5 different mice were analyzed). Data are reported±S.D. *P<0.05

In order to clarify the cellular mechanism by which the transgene expression promotes cardiac repair, we investigated the growth and differentiation of c-Kit^TgD814Y^-expressing cells after CI. Immunofluorescence analysis was performed to identify total cardiac c-Kit^+^ cells internal to the injured area 9 days after injury (Figure 5a). At this time point, c-Kit^+^ cell number that showed an evident elongated shape, was higher in transgenic mice compared with wt mice (Figure 5b; 72±9 cells/mm^2^ and 55±7 cells/mm^2^). To verify the vascular lineage commitment rate of total c-Kit^+^ cells, we assessed the expression of c-Kit together with endothelial (CD31) and *α*-smooth muscle actin lineage markers within the damaged area. c-Kit^+^ cells were practically negative for smooth muscle actin in both genotypes, whereas more than 90% of c-Kit^+^ cells co-stained with CD31 in *c-Kit*^*TgD814Y*^ mice compared with ~60% in wt mice (Figures 5c and d). Concurrently, at 30 days after CI, an increased capillary density was observed in transgenic mice compared with wt mice in peri-damaged area (Figure 5e). Finally, transgenic and wt mice were implanted with mini-osmotic pumps to systemically release BrdU immediately after injury for 28 days, in order to follow myocardial cell regeneration. A threefold increase of newly, mono-nucleated-formed BrdU^+^ cardiomyocytes was observed in transgenic hearts when compared with wt mice at 1 month after myocardial necrosis by CI (Figure 5f). Altogether, these data suggest that, *in vivo*, constitutive c-Kit receptor activation in myocardial cells fosters cardiac repair increasing angiogenic and myogenic response to injury.

### c-Kit receptor activation increases CSC activation and differentiation *in vitro*

**Figure 6:**
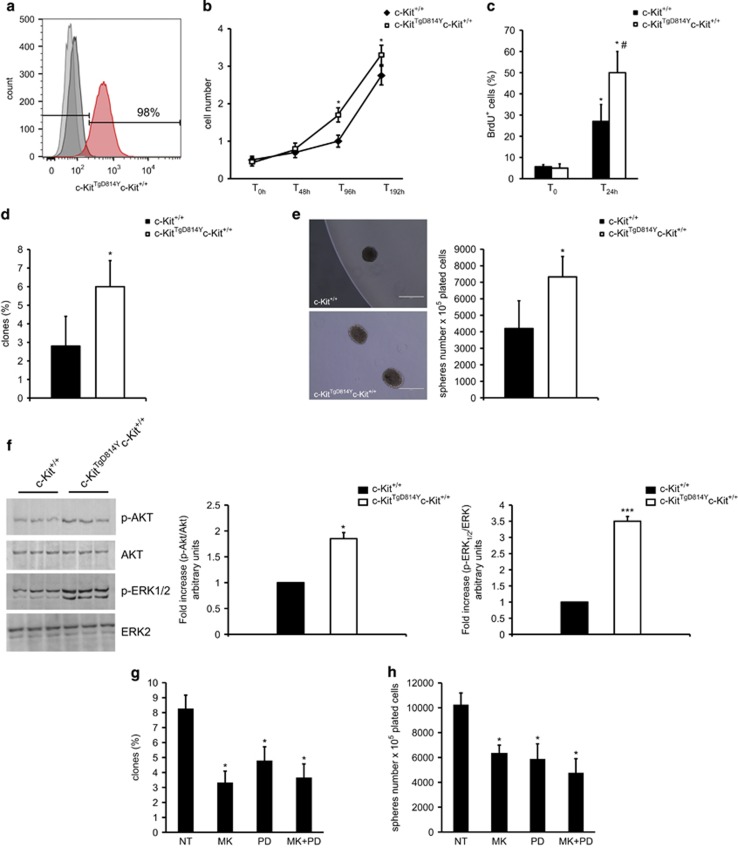
CSCs are activated by *c-Kit*^*TgD814Y*^. (**a**) Flow cytometry of CSCs isolated from *c-Kit*^*+/+*^ and *c-Kit*^*TgD814Y*^ hearts. (**b** and **c**) Cell growth curve and BrdU incorporation evaluation of *c-Kit*^*+/+*^ and *c-Kit*^*TgD814Y*^ CSCs. Data are reported±S.D. *P<0.05. (**d** and **e**) Clone and sphere formation assay was performed on *c-Kit*^*+/+*^ and *c-Kit*^*TgD814Y*^ CSCs and the results are summarized in the histogram bar. Representative images of clones is shown. Data are reported±S.D. *P<0.05. (**f**) WB and densitometric analysis on protein extracts from *c-Kit*^*TgD814Y*^ and *c-Kit*^+/+^ CSCs of three different experiments. (**g** and **h**) Clone and sphere formation assay was performed on *c-Kit*^*TgD814Y*^ CSCs after 14 days of treatment with AKT (MK2206, 1 *μ*M), ERK inhibitor (PD98059, 100 *μ*M) or the combination of them. Data are reported±S.D. *P<0.05 *versus* untreated sample (NT)

The data shown above cannot unequivocally establish a direct precursor-to-differentiated cell product relationship between committed c-Kit^+^ cells and the increased formation of endothelial cells and cardiomyocytes in transgenic mice after injury. Thus, to directly test this hypothesis, we isolated CD45^−^c-Kit^+^ CSCs^35^ from adult *c-Kit*^*TgD814Y*^ transgenic and wt mice (Figure 6a). When plated in LIF-conditioned CSC medium, transgenic c-Kit^TgD814Y^ cells showed an increased cell growth when compared with wt CSCs as demonstrated by their growth kinetic curve and BrdU incorporation rate *in vitro* (Figures 6b and c). This higher growth potential was correlated with an increased clonogenic potential established by single-cell deposition (Figure 6d). Furthermore, constitutive c-Kit receptor activation promoted CSC sphere formation, an *in vitro* marker of multipotent cells, when compared with wt CSCs (Figure 6e). These cellular characteristics of c-Kit^TgD814Y^ CSCs were associated with c-Kit receptor activation as demonstrated by an increased ERK1/2 and AKT phosphorylation in transgenic *versus* wt CSCs (Figure 6f). Indeed, AKT inhibition by MK2206, a known selective allosteric inhibitor of AKT, and ERK1/2 inhibition by PD98059, a small molecule selectively targeting ERK1/2, were both able to reduce clonogenesis and spherogenesis rate in transgenic c-Kit^TgD814Y^ CSCs (Figures 6g and h).

**Figure 7:**
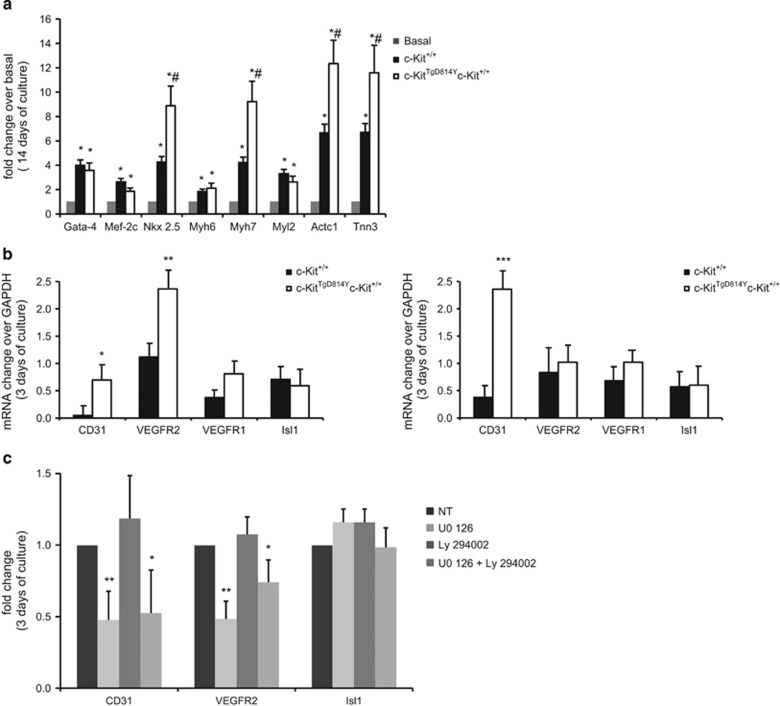
Enhanced endothelial differentiation of *c-Kit*^*TgD814Y*^ CSCs. (**a**) qRT-PCR on *c-Kit*^*TgD814Y*^ and *c-Kit*^+/+^ CSCs for cardiac marker after switching the cells into cardiomyocytes differentiating medium. The data are expressed as fold increase over the basal. Data are reported±S.D. *P<0.05 *versus* basal and ^#^*P*<0.05 *versus c-Kit*^+/+^. (**b**) Semiquantitative RT-PCR quantification of endothelial markers 3 and 10 days after switching *c-Kit*^*TgD814Y*^ and *c-Kit*^+/+^ CSCs into endothelial medium. Results are expressed as fold increase over GAPDH±S.D. *P<0.05, **P<0.01 and ***P<0.001. (**c**) Endothelial differentiation was inhibited by 72 h treatment with ERK1/2 inhibitor (U0, 10 *μ*M) but not with AKT inhibitor (LY, 10 *μ*M). Data are reported as mRNA fold change±S.D. *versus* NT, *P<0.05, **P<0.01

When cells were primed in LIF-deprived low-serum (2%) cardiomyogenic condition, CSCs from c-Kit^TgD814Y^ mice show an increased myogenic specification when compared with wt CSCs. Indeed, an increased number of mRNA transcripts for Nkx2.5, known cardiomyocyte transcription factor and for Myh7, Actc1 and Tnn3, main contractile genes were detected in c-Kit^TgD814Y^ compared with wt CSCs (Figure 7a). Both MK2206 and PD98059 treatment significantly affected cell viability in low-serum myogenic differentiation conditions, preventing myogenic specification with negligible upregulation of the above cardiac transcription and contractile gene mRNAs (data not shown).

On the other hand, when CSCs were plated in specific LIF-deprived endothelial differentiation medium, a significant increase of CD31 and VEGFR2 endothelial RNA transcripts was observed in c-Kit^TgD814Y^ compared with c-Kit^+/+^ CSCs already at 3 days in differentiating medium, which was followed at 10 days by a higher number of CD31^+^ endothelial differentiated cells (Figure 7b and Supplementary Figure 4A). Interestingly, endothelial differentiation was abrogated by incubation with MAPK inhibitor (UO), but not with AKT inhibitor (LY) (Figure 7c and Supplementary Figure 4B), suggesting that MAPK is necessary for c-Kit-stimulated endothelial lineage differentiation.

Overall these data show that, *in vitro*, c-Kit receptor constitutive activation exerts several beneficial effects on CSCs enhancing their amplification, when primed to proliferate, and promoting their cardiomyocyte and endothelial differentiation, when coaxed to specific cardiac cell commitment.

## Discussion

In the present study, a new mouse model was generated expressing c-Kit receptor with an activating amino acidic substitution to study the expression and activation of the receptor in cardiac development and its role during regenerative processes. The transgenic mice were generated mutating a BAC vector containing endogenous regulatory sequences of 67 Kb upstream the starting codon and 58 Kb downstream the stop codon. As previously described for other transgenic mice generated with vectors spanning similar regions of the *c-Kit* locus,^27, 36^ transgene expression was not detected in fetal liver and adult bone marrow, probably owing to the lack of enhancer regions in the regulative sequence of the BAC,^27^ but its expression and activation was increased up to twofold compared with wt in heart other than in cerebellum and testis. In this way, it was possible to study for the first time the receptor activity physiologically expressed in cardiac tissue and to clarify the controversial findings reported on the putative contribution of bone marrow *versus* cardiac resident c-Kit^+^ cells in heart repair.^18, 22, 23, 37^

Wild-type c-Kit^+^ cells respond to c-Kit cognate ligand by increasing ERK1/2 and AKT phosphorylation.^38, 39^ We interestingly found a consistent increase of ERK1/2 and AKT phosphorylation levels in hearts and isolated cardiac cell preparations obtained at different ages of transgenic mice. This level of activation, specific to the activating gene mutation and confirmed by protein analysis of *c-Kit*^*TgWT*^ mice, was not further stimulated by treating the cells with the specific c-Kit ligand.

Altogether, these results support and strongly confirm that ERK1/2 and AKT are among the major targets of c-Kit receptor in cardiac c-Kit^+^ cells. Indeed, the substitution of the aspartic acid 814 with tyrosine was able to recapitulate the activation of PI3K and MAPK pathways naturally occurring following KL stimulation of the wt receptor, thus allowing to investigate the physiological role of c-Kit receptor in the heart. The 814 Aspartic mutation was previously found in different type of tumors and it was reported to induce a constitutive activation of c-Kit.^24, 25, 26^ Notably, transgenic mice carrying *c-Kit*^*TgD814Y*^ mutation were monitored for more than 2 years and no tumors such as GIST, seminoma or mastocytosis were detected.

A fully characterization of c-Kit^TgD814Y^ expression in the heart was performed by WB and immunohistochemistry showing that the activated receptor is expressed during embryogenesis, neonatal and adult life. BAC vectors, preserving endogenous gene regulation, gave the opportunity to study cardiac c-Kit^+^ cells in respect of their putative origin from the first heart field, proepicardium or epithelial to mesenchymal transition. Further characterization is required to verify if the two subpopulations expressing low and high c-Kit levels, recently described,^40, 41, 42, 43^ are differently regulated in hearts of *c-Kit*^*TgD814Y*^ mice. By crossing the *c-Kit*^*TgD814Y*^ mice to *pKit*^*EGFP*^ mice, we concluded that number and localization of c-Kit^+^EGFP^+^ double positive cells were similar between *pKit*^*EGFP*^ and *c-Kit*^*TgD814Y*^*pKit*^*EGFP*^ hearts indicating a similar behavior of wt and transgenic cells in the intact organ. These observations suggest that the constitutive activation of ERK1/2 and AKT pathways *in vivo* does not influence proliferation and/or cell death of these cells in normal conditions or that these events occur in a narrow window before c-Kit^+^ cells undergo differentiation. On the other hand, *in vitro* experiments showed a better proliferation and growth potential of c-Kit^TgD814Y^ CSCs purified from adult c-Kit^*TgD814Y*^ mice indicating that the mutation of the receptor is by itself capable to fully activate the proliferation program of these cells.

To test the efficacy of the transgene in heart regeneration, we took advantage of the CI technique.^44, 45^ CI was chosen because it is able to induce a focal, transmural and reproducible heart damage. Transgenic mice were more resistant to the injury (Figure 4). All mice survived after the CI and showed cardiac tissue repair within 1 month after damage, suggesting a protective role by the c-Kit^TgD814Y^ transgene.

Moreover, a consistent reduction of fibrotic area was observed after CI in transgenic mice (Figure 4f). Nine days after the CI the number of c-Kit^+^ cells was increased in the damaged area of *c-Kit*^*TgD814Y*^ mice compared with *c-Kit*^*+/+*^ mice (Figure 5b). Because it has been reported that ERK1/2 and AKT phosphorylation might induce migration and/or differentiation of stem cells,^17, 39, 46^ our findings indicate that these mechanisms might also occur following CI in *c-Kit*^*TgD814Y*^ mice. This hypothesis is in line with the observation of numerous elongated c-Kit^+^ cells in the damaged area which express the endothelial CD31 marker and with the higher capillary density found in peri-damaged area of transgenic hearts, allowing to speculate that c-Kit activation is required for novel vessel formation during the early stages of heart repair. These results are in line with the recent findings showing that c-Kit^+^ cells are committed to the endothelial differentiation^42, 47, 48^ and, for the first time demonstrate that activation of MAPK but not AKT signaling pathway is fundamental for c-Kit-mediated endothelial differentiation. Moreover, the results obtained with BrdU incorporation also suggest a role of c-Kit^TgD814Y^ receptor in myocytes regeneration, following heart injury favoring cardiomyocyte turnover for heart repair. The *in vivo* bi-potent fate of c-Kit^TgD814Y^ precursors to support both endothelial and cardiomyocyte differentiation was confirmed *in vitro* by purifying and selectively culturing c-Kit^TgD814Y^ CSCs and showing that both MAPK and AKT pathways are required for CSC growth and clonogenic properties as well as for their endothelial and myogenic commitment. Overall, these results highlight the crucial role of c-Kit receptor activation in heart repair with distinct involvement of AKT and/or ERK1/2 signaling pathways to mediate c-Kit functions in both healthy and injured hearts.

## Materials and Methods

### Mice generation

The generation of *c-Kit*^*TgD814Y*^ transgenic mice was obtained by substituting G to T 2468 in the *c-Kit* gene of the murine BAC RP23-309C11 through homologous recombination as previously described.^49, 50^

The murine BAC RP23-309C11, containing 80 kb of *c-Kit* genome sequence and 68 kb of sequence upstream and 57 kb of sequence downstream the *c-Kit* initiation and stop codons, respectively, was engineered substituting the G to T 2468 to generate *c-Kit*^*TgD814Y*^ transgenic mice. Electrocompetent DH10B bacteria with the BAC RP23-309C11 were electroporated with 100 bp oligos containing complementary 20 bp mutated regions around the G to T 2468 nucleotide responsible for the switch of aspartic to tyrosine amino acid. Following the first homologous recombination driven by a temperature-inducible mini-*λ* phage, a second electroporation, was performed using a 100 bp oligos pairs with the G to T 2468 substitution surrounded with endogenous *c-Kit* sequence to replace the first oligos pairs and leave the single point mutation. Following the second homologous recombination, the mutation was inserted in the BAC RP23-309C11. The G to T 2468 mutation was confirmed by sequencing analysis and the purified BAC was microinjected into the pronuclei of fertilized C57BL/6 eggs by standard methods. Founder lines were identified by RT-PCR analysis using Sp6 For (5′-ATTTAGGTGACACTATAG-3′) and 309 Rev (5′-CTCTTCTTGACAGTTCCTGC-3′) and CM for (5′-TGTTCACCCTTGTTACACCG-3′) and CM Rev (5′-CCACTCATCGCAGTACTGTT-3′) primers designed in between the vector and *c-Kit* gene and in the chloramphenicol resistance cassette, respectively.

Two transgenic founders lines, Tg7 and Tg8, were maintained by breeding them to C57BL/6 mice. Homozygotes *pKit*^*EGFP*^(gifted by Prof Ottolenghi,^31^) were crossed with *c-Kit*^*TgD814Y*^*c-Kit*^*+/+*^ to generate *c-Kit*^*TgD814Y*^*c-Kit*^*+/+*^*pKit*^*EGFP*^mice (referred as *c-Kit*^*TgD814Y*^*pKit*^*EGFP*^).

Heterozygote *c-Kit*^*+*^^*/−*^ mice were generated by replacement of the 16^th^ intron with a PGK-Neo cassette through homologous recombination, resulting in functional inactivation of the gene. A 7 kb BamH1 fragment of the mouse c-Kit gene spanning from intron 14 to intron 17 was utilized to generate by PCR amplification a 1.5 Kb left arm of homology (spanning from intron 14 to exon 16, with an artificial 3′ *Xho*I site) and a 4.5 Kb right arm of homology (spanning from exon 17 to intron 17, with an artificial 5′ EcoRV site). A *Xho*I-EcoRV 1.6 Kb PGK-Neo cassette was added upstream to the EcoRV site of the right arm, and a *Sal*I 3.0 Kb PGK-TK cassette was added to the 3′ *Bam*H1 site (after partial filling of the *Sal*I site in the cassette and the *Bam*H1 site in the right arm). Subsequently, the left arm of homology was added to the *Xho*I site of the construct. A 11 Kb *Not*I gene targeting fragment (containing sequentially the left arm of homology, the PGK-Neo cassette, the right arm of homology and the PGK-TK cassette, see Supplementary Figure 1) was electroporated in ES cells, which were subsequently treated with neomycin and gancyclovir for positive and negative selection, respectively. Screening of homologous recombinant ES colonies was performed by Southern blot using a 600 bp EcoRV-*Bam*H1 external 5′ genomic c-Kit probe and digesting genomic DNA with EcoRV. Positive recombinants were identified by the appearance of a 3 Kb diagnostic band. The identified recombinant ES colonies were injected in mouse blastocysts by standard methods to generate heterozygous mice in which the 1 kb 16th c-Kit intron had been replaced by the 1.6 Kb PGK-Neo cassette. Heterozygote *c-Kit*^*+/−*^ mice were crossed with *c-Kit*^*TgD814Y*^*c-Kit*^*+/−*^ to generate *c-Kit*^*TgD814Y*^*c-Kit*^*+/+*^, *c-Kit*^*TgD814Y*^*c-Kit*^*+/−*^ or *c-Kit*^*TgD814Y*^*c-Kit*^*−/−*^ mice. These mice showed all the typical phenotypic aspects of the White spotting mutation, including lethality in the homozygous condition, are in agreement with previous evidence that the inclusion of a *neo* gene in intronic sequences severely interferes with the expression of the *c-Kit* gene.^51^

Mice of 1–9 dpp and adults were killed by cervical dislocation. All our investigations conform with the Directive 2010/63/EU of the European Parliament on the protection of animals used for scientific purposes, and were conducted with the approval of the Tor Vergata University's Animal Use for Research Ethic Committee and by the Italian Ministry of Health.

### Copy number assay

In order to quantify *c-Kit*^*TgD814Y*^ transgene copy number, we performed a real-time PCR assay, based on evaluation of genomic c-Kit sequence from the transgene. Probe and primers were specific for exon 14 of the c-Kit gene.

The *c-Kit*^*TgD814Y*^ copy number was determined using TaqMan Copy Number Assays (Life Technologies, Thermo Fisher Scientific, Waltham, MA, USA) according to the manufacturer's instructions. In brief, genomic DNA (20 ng) was combined with 2 × TaqMan Genotyping Master Mix, TaqMan Copy Number Assay for mouse c-Kit (Mm00161478_cn Cat. # 4400291) and TaqMan Copy Number Reference Assay for mouse Tert in a 20 *μ*l reaction volume. The assay was performed using the Applied Biosystems I 7500 Fast Real-Time PCR System (Life Technologies, Thermo Fisher Scientific) and the following thermal cycling conditions: 95 °C for 10 min, and 40 cycles of 95 °C for 15 s and 60 °C for 1 min. Samples were assayed using triplicate wells for each gene of interest and copy numbers were estimated by relative quantitation normalized to the known copy number of the reference sequence using the comparative Ct (ΔΔCt) method. The Ct data were subsequently compared with a calibrator sample known to have two copies of the target sequence, analyzed by Applied Biosystems CopyCaller Software (v.2.0; Applied Biosystems) according to the product instruction. Each analysis was carried out with three mice tail DNA of *c-Kit*^*+/+*^ and *c-Kit*^*TgD814Y*^*c-Kit*^*+/+*^ mice. The analysis indicates that transgenic mice integrated one copy of BAC RP23-309C11 construct in genomic DNA. ***P<0.001. The results were accepted only when calling confidence was >80% and ΔCq standard deviation between replicates was <0.20.

### Newborn cardiac cells isolation, stimulation and immunofluorescence

Cardiac cells were prepared from ventricles of 1–3 dpp newborn pups and cultured as previously described.^52^

Freshly isolated newborn cardiac cells, pretreated or not for 30 min with 5  *μ*M Imatinib-mesylate (sc-202180, Santa Cruz Biotechnology, Inc., Dallas, TX, USA), were stimulated for 10 min with 100 ng/ml KL (R&D Systems, Inc., Minneapolis, MN, USA) before protein extraction.

Immunofluorescence on newborn neonatal myocytes was performed after 24 h of culture. Cells were fixed with 4% PFA, blocked with PBS/0.5% BSA/3% horse serum and stained overnight at 4 °C with mouse anti-sarcomeric myosin (1:5; MF20, Hybridoma Bank, Iowa, IA, USA); rabbit anti-MEF2C (1:100; Abcam: ab64644, Cambridge, UK) and goat anti-c-Kit (1:100; R&D) and then incubated 1 h at 37 °C with anti-rabbit FITC and anti-goat Cy5 secondary antibodies (1:300; Jackson ImmunoResearch Laboratories, Inc., West Grove, PA, USA). Images were acquired by Nikon Eclipse Ti - S microscope (Nikon Instruments S.p.A, Firenze, Italy).

### Adult CSC purification and differentiation

CSCs were isolated from adult transgenic and wt hearts by enzymatic methods.^35, 40^ For CD45^−^c-Kit^+^ cell purification, myocyte-depleted small cardiac cells were incubated with microbeads conjugated with anti-mouse CD45 antibody (Miltenyi Biotec S.r.l., Calderara di Reno (BO), Italy) and removed from the preparation by magnetic-activated cell sorting (Miltenyi). From the CD45^−^ fraction, the c-Kit^+^ (CD45^−^) cardiac cells were enriched through incubation with a microbeads-conjugated mouse monoclonal antibody against c-Kit (Miltenyi) and separated by magnetic-activated cell sorting.

Freshly isolated CD45^−^c-Kit^+^ cardiac cells were cultured on gelatin-coated dishes in CSC growth medium^35, 40^ before clonogenic, spherogenic and BrdU assays.

Cardiosphere myogenic differentiation was performed as previously described.^35^

Endothelial differentiation was obtained by culturing the CSC for 3–10 days in MEM Alpha (Life Technologies), 10% ESQ-FBS (Life Technologies), 1 *μ*M dexamethasone, 50 *μ*M/ml ascorbic acid, 10 mM *β*-glycerophosphate (all from Sigma-Aldrich, Milano, Italy) and 10 ng/ml VEGF (PeproTech EC Ltd., London, UK). LY294002 (10 *μ*M, Calbiochem, San Diego, CA, USA) and UO126 (10 *μ*M, Cell Signalling Technology, Danvers, MA, USA) were added to the culture for AKT and ERK1/2 inhibition.

### Clonogenic, spherogenic and BrdU assays

To show clonogenicity, single-cell cloning was used through depositing single CD45^−^c-Kit^+^ cardiac cell into wells of 96-well gelatin-coated Terasaki plates by serial dilution. Individual CD45^−^c-Kit^+^ cells were grown in mCSFM for 1–3 weeks when clones were identified.^35, 40^ The clonogenicity of the CD45^−^c-Kit^+^ cells was determined by counting the number of wells in each 96-well plate, which contained clones and expressed as a percentage. A total of 10 plates were analyzed for each experiment.

For cardiosphere generation, CD45^−^c-Kit^+^ CSCs were placed in bacteriological dishes with cardiosphere generation medium (mCSFM) composed of 1:1 ratio of CSC growth medium and Neural Basal Media supplemented with B27 and N2 supplements (Life Tech). Cardiospheres were counted per plate at 14 days and the number expressed as a percentage relative to the number of plated CSCs.^35, 40^ For Akt inhibition, MK2206 (Enzo Biochem, New York, NY, USA) was used at a concentration of 1 *μ*M. For Erk1/2 inhibition, PD98059 (Cell Signaling) was used at a concentration of 100 *μ*M.

To assess the proliferative activity of c-Kit^+^CD45^−^ cardiac cells *in vitro*, 10 *μ*M bromodeoxyuridine (BrdU, Roche, Monza, Italy) was administered in cell plate for 30 min after 48 h serum-free medium conditions (T0) and every 8 h for 1 day. Then, cells were analyzed by flow cytometry (PE-conjugated Anti-BrdU, eBiosciences, San Diego, CA, USA).

### Bone marrow and blood cells isolation

Bone marrow cells were isolated from adult mice by flushing both femurs and tibias with 2 ml of PBS using a 25-gauge needle syringe. Cells were pelleted, washed and re-suspended for the analysis.

For white blood inflammatory and remodeling marker staining, 200 *μ*l of blood samples were collected from mice eyes in heparin-treated vials then red blood cells were lysated using RBC Lysis Buffer (BioLegend, London, UK) as the protocol.

### Flow cytometry analysis

Bone marrow cells were re-suspended in FACS buffer (PBS/2% FBS/2 mM EDTA) and stained for 1 h at 4 °C with rat anti-c-Kit CD117 Alexa Fluor 647 (Invitrogen: RM6221, Thermo Fisher Scientific-Life technologies, Waltham, MA, USA) antibody. Dead cells were stained with 7 Amino-Actinomycin D dye (Sigma Aldrich).

White blood cells were stained in 100 *μ*l of FACS buffer with rat anti-CD11b PE-Cy7 (Pharmingen: 25-0112, Gurgaon, Haryana, India) and rat anti-GR-1 APC antibodies (Pharmingen: 17-5931) at 4 °C for 1 h. Dead cells were stained with Sytox-Blue dye. Analysis was performed with CyAn cytofluorimeter (Dako, Milano, Italy).

Isolated CSC analysis was performed on FACSCanto II with FlowJo software to identify c-Kit expression by mouse monoclonal APC-conjugated anti-c-Kit antibody (Miltenyi). Appropriate labeled isotype controls were used to define the specific gates.

### mRNA extraction and RT-PCR analyses

mRNA was extracted from CSCs with Trizol (Sigma Aldrich) using Qiagen RNAEasy miniKit (Milano, Italy) following the manufacturer's instructions. Traces of genomic DNA were removed before retro-transcription using DNA Free Zymo Research kit. RNA was quantitated using a Nanodrop 2000 Spectrophotometer (Thermo Fisher Scientific, Waltham, MA, USA) and 0.5–1.0 *μ*g of total RNA was retro-transcribed using SuperScript II transcriptase (Life Technologies/Invitrogen/Thermo Fisher Scientific) according to the manufacturer's instructions using the High Capacity cDNA Kit (Applied Biosystems).

Reverse transcription-qPCR was performed with the TaqMan Primer/Probe sets (specific primers were purchased from Invitrogen and the relative catalog numbers are available upon request) using StepOne Plus Real Time PCR System (Applied Biosystems) following the manufacturer's instruction.

Data were processed by the ΔCt method using StepOne Software v2.3. mRNA was normalized with GAPDH and all reactions were carried out in triplicate.

For semiquantitative RT-PCR, primers were as follows: CD31 forward 5′- CGAAGTTAGAGTTCTCCTCC-3′ and CD31 reverse 5′- TCTGATACTGCGACAAGACC-3′ VEGFR1 forward, 5′-GGTATGACTTCTGCACTGAG-3′ and VEGFR1 reverse 5′- CACCAATGTGCTAACCGTCT-3′ VEGFR2 forward, 5′-GTGTCTCTTTGCGCTAGGTA-3′ and VEGFR2 reverse, 5′-TTGCCTCACAGAAGACCATG-3′

Isl1 forward, 5′-GCAGCAACCCAACGACAAAA-3′ and Isl1 reverse, 5′-AATTGACCAGTTGCTGAAAAGC-3′ GAPDH forward 5′-GTGAAGGTCGGTGTGAACG-3′ GAPDH reverse 5′- ATTTGATGTTAGTGGGGTCTCG - 3′. RT-PCR products were separated in a 1% agarose gel stained with ethidium bromide.

### Immunoprecipitation and WB analyses

Heart tissue, newborn cardiac cells and CSCs were homogenized into RIPA buffer (150 mmol/l NaCl, 50 mmol/l Tris-HCl pH 7.6, 1% NP40, 0.5% Sodium deoxycholate, 0.1% Sodium dodecyl sulfate, 10 mmol/l *β*-glicerophosphate, 1mmol/l DTT) containing phosphatase and protease inhibitors before WB analysis as described in Supplementary Methods.

For immunoprecipitation analyses, specific antibodies were incubated with a mixture of protein A and/or G-Sepharose beads (Sigma-Aldrich) in lysis buffer containing 0.05% BSA for 60 min under constant shaking at 4 °C. The beads were then washed twice with lysis buffer and incubated for 90 min at 4 °C with clarified cell lysates under constant shaking. Sepharose beads-bound immunocomplexes were rinsed three times with lysis buffer and eluted with Laemmli buffer. Proteins were separated on SDS-PAGE 8% gels and transferred to PVDF membranes (GE Healthcare, Milano, Italy). These were incubated overnight at 4 °C with rabbit anti-c-Kit (1:100) sc-168; mouse anti-pERK1/2 (1:1000) sc-7383; rabbit anti-ERK 2 (1:1000) sc-154; rabbit anti-pAKT1/2/3-ser473 (1:1000) sc-7985; mouse anti-AKT1/2/3 (1:1000) sc-81434; mouse anti-pTyr (1:1000) Millipore 05-321 (Vimodrone (MI), Italy); mouse anti-tubulin (1:2000) Sigma T 5168 all from Santa Cruz Biotechnology and then with the appropriate horseradish peroxidase-conjugated secondary antibody (Santa Cruz Biotechnology). The horseradish peroxidase conjugate was detected by chemiluminescence with an ECL Kit (Santa Cruz Biotechnology) and autoradiography. Densitometry was performed using Molecular Dynamics Densitometer and ImageJ software.

Protein lysates obtained from CSCs (~50 *μ*g) were separated on 10% SDS-polyacrylamide gels. After electrophoresis, proteins were transferred onto nitrocellulose filters, blocked with either 5% dry milk or 5% bovine serum albumin, and incubated with Abs against AKT (#9272), p-AKT (#4058 S), ERK1/2 (#9102) and pERK1/2 (#9101) (all from Cell Signaling) at dilutions suggested by the manufacturers. Proteins were detected by chemiluminescence using horseradish peroxidase-conjugated 2Abs and the Alliance UVITEC Cambridge system (Eppendorf, Milano, Italy).

### Heart injury

The CI was performed by a technician blinded to the genotype of mice, essentially as previously reported.^45^ Briefly, under general anesthesia with Avertin (Sigma, 250 mg/kg), mice were subjected to surgery. The abdominal cavity was opened and CI was inflicted through the intact diaphragm on the heart pushed toward the probe. Heart damage was performed by applying for 5 s a 5 mm diameter metal probe cooled to −196 °C with liquid nitrogen. The abdominal cavity was closed with sutures. Mice received 500 *μ*l of glucose solution (5% glucose in physiologic solution) and the analgesic Atradol (3 mg/kg), soon after the surgery. Mice were killed and their hearts harvested 3 (*c-Kit*^*+/+*^ *n*=2; *c-Kit*^*TgD814Y*^*c-Kit*^*+/+*^ *n*=2), 9 (*c-Kit*^*+/+*^ *n*=10; *c-Kit*^*TgD814Y*^*c-Kit*^*+/+*^ *n*=8) and 30 (*c-Kit*^*+/+*^ *n*=12 and *c-Kit*^*TgD814Y*^*c-Kit*^*+/+*^ *n*=13) days after the CI.

A set of mice (*c-Kit*^*+/+*^ *n*=10; *c-Kit*^*TgD814Y*^*c-Kit*^*+/+*^ *n*=10) were treated with BrdU for 30 days. Half of them, for each genotype, was damaged with CI as previously described. BrdU (0.6 M) was administered continuously using Alzet osmotic mini pumps (Charles River Laboratories, CALCO (Lecco), Italy). The latter were implanted subcutaneously in the dorsal region via a small interscapular incision using sterile surgical technique, while the animals were under light avertin anesthesia.

### Tissue harvesting, immunofluorescence, immunohistochemistry and TUNEL assay

Hearts were collected at different ages, the atria were removed and the ventricles were placed in Hank's balanced solution (Euroclone, Pero (MI), Italy) squeezing gently to remove the remaining blood from the chambers. After washing in PBS, the ventricles were embedded in OCT (Bio-Optica, Milano, Italy), frozen in isopentane chilled with liquid nitrogen and stored at −80 °C before immunohistochemistry and immunofluorescence analyses as described in Supplementary Methods. In order to preserve GFP, ventricles taken from *pKit*^*EGFP*^ mice and *c-Kit*^*TgD814Y*^*pKit*^*EGFP*^ mice were pre-fixed in PBS/4% PFA pH 7.4 (Sigma) overnight at 4 °C and then included in OCT. Embryos were directly included in OCT, frozen in isopentane chilled with liquid nitrogen and stored at −80 °C.

For EGFP^+^ cardiac cell count, at least 20 sections of 5 *μ*m thickness were collected from each heart and analyzed for c-Kit and EGFP staining at different postpartum developmental time for *pKit*^*EGFP*^ mice and *c-Kit*^*TgD814Y*^*pKit*^*EGFP*^ mice: at 1 day *n*=4 and *n*=5 mice, respectively; at 10 days *n*=4 and *n*=6 mice, respectively; at 2 months old *n*= 5 and *n*=6 mice, respectively. Images were acquired with Zeiss microscope (Axioskop 2 plus, Zeiss, Carl Zeiss Microscopy, Thornwood, NY, USA). EGFP-positive cardiac cells were counted throughout the left and right ventricle for each section. The EGFP-positive cell number was calculated as mean number per section.

Immunofluorescence was performed in 5 *μ*m-thick sections fixed in PBS/4% PFA for 10 min at room temperature before staining. After permeabilization and blocking with PBS/1% BSA, slices were incubated with primary antibodies diluted in PBS/1% BSA overnight at 4 °C. Undamaged and damaged heart sections were stained with the following primary antibodies: goat anti-c-Kit (R&D) 1:100 and rat anti-CD31 (eBioscience: 14-0311) 1:100; rabbit anti-*α*-SMA (Abcam: ab5694) 1:100. In order to reveal c-Kit receptor in embryos, sections were stained with goat anti-c-Kit (R&D) diluted 1:50. Secondary antibodies anti-rat Alexa Fluor 488, anti-rabbit FITC and anti-goat Cy5 all from Jackson were diluted 1:300. Nuclei were stained by Hoechst 33342 solution.

To quantify fibrotic tissue, trasversal serial sections of ventricles (5 *μ*m of thickness) were collected, fixed in Bouin solution (Sigma) and stained with Masson's Trichrome kit (Sigma). Collagen was detected in blue and myocardial cells in red. Images were acquired by Axioskop 2 plus, Zeiss microscope and ImageJ software was used to quantify the fibrotic area. The fibrotic residual area of each collected section was measured in pixels and expressed as percentage respect to total area, without the lumen. The injured size was then calculated as the mean percentage of slices for all ventricles.

Apoptosis was evaluated on three heart sections derived from three different *c-Kit*^*+/+*^ and *c-Kit*^*TgD814Y*^ *c-Kit*^*+/+*^mice 9 days after the CI by following TUNEL assay (Roche) manufacturer's instructions.

### BrdU, WGA and IB-4 stainings

Damaged hearts from BrdU-treated mice were perfused with buffered formalin and embedded in paraffin, and 5 *μ*m-thick sections were cut.

Antigen retrieval was achieved using Target Retrieval Solution, Citrate pH 6 (DAKO). Myocyte cytoplasm was detected using an antibody against *α*-actinin (1:50 dilution, Clone H-300, Santa Cruz Biotechnology) for 3 h at 37 °C and this was detected with anti-rabbit Alexa Fluor 647 (1:100 dilution; Jackson Immunoresearch Laboratories, Inc., West Grove, PA, USA). BrdU was detected using an antibody against BrdU (1:50 dilution; Roche) for 45 min at 37 °C. This antibody was detected with an anti-mouse Alexa Fluor 488 (1:100 dilution; Jackson Immunoresearch). Newly formed myocytes were detected through double staining for BrdU and *α*-actinin. Secondary antibody incubation was carried out at 37 °C for 1 h. Wheat germ agglutinin (Alexa Fluor 594 conjugate WGA; Life Technologies) staining was performed for cardiomyocyte dimension analysis. The nuclei were counterstained with the DNA-binding dye, 4, 6-diamidino-2-phenylindole (Sigma) at 1 *μ*g/ml. Sections were mounted in Vectashield and analyzed and scanned using confocal microscopy (Leica Microsystems TCS SP5, Milano, Italy). ImageJ software was used to quantify the cardiomyocyte area.

For IB-4 staining, 5 *μ*m heart sections were deparaffinized and treated with 10 mM sodium citrate solution (Santa Cruz Biotechnology), pH 6.0, in microwave for 10 min. After permeabilization with 0.1% PBS-Triton X-100 v/v at RT for 8 min, sections were blocked with PBS-1% BSA w/v for 30 min at RT and capillaries were stained with IB-4 - FITC conjugated (Sigma) overnight at 4 °C. Nuclei were stained with Hoechst 33342. Slides were mounted with glycerol in 50 mM Tris-HCl solution pH 8.7 (1:1). Images were acquired by Nikon Eclipse Ti - S microscope. The number of capillary was counted in 10 images/section and reported as fold change.

### Statistical analyses

Statistical analysis was performed with GraphPad Prism version 6.00 for Macintosh (GraphPad Software, Inc., La Jolla, CA, USA). Quantitative data are reported as mean±S.E. or mean±S.D. and binary data by counts. Significance between two groups was determined by Student's *t* test or paired *t* test as appropriate. For comparison between multiple groups, ANOVA was used. A *P* value <0.05 was considered significant. Bonferroni *post hoc* method was used to locate the differences. In these cases, the type 1 error (*α*=0.05) was corrected by the number of statistical comparisons performed. If not specified, a *n*=4 sample size was used for the *in vitro* cell and molecular biology experiments. The Kruskal–Wallis (for multiple-group comparison) and the Mann–Whitney U tests (for comparison between two groups) were performed.

## Abbreviations

CI: cryoinjury
CSCs: cardiac stem cells
IB-4: isolectin B4
KL: c-Kit ligand
