# Supplementary material for: Activated c-Kit receptor in the heart promotes cardiac repair and regeneration after injury
Source: Cell Death Dis. 2016 Jul 28;7(7):e2317–. doi: 10.1038/cddis.2016.205 (PMC4973348; doi:10.1038/cddis.2016.205)
Supplement: Supplementary Information [file cddis2016205x1.pdf]

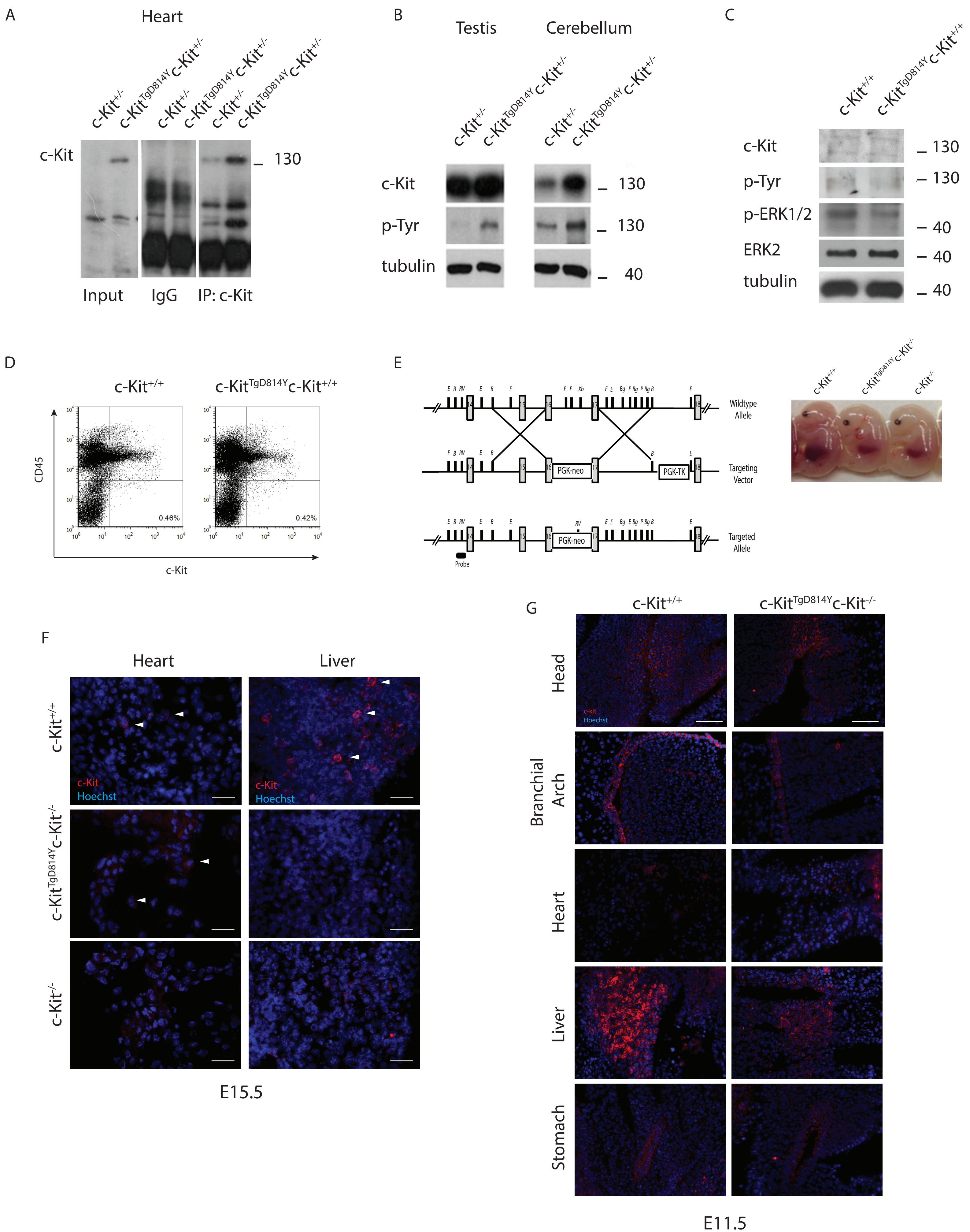
A
Heart
c-Kit+/-
c-KitTgD814Y c-Kit+/-
c-Kit
130
Input
IgG
IP: c-Kit
B
Testis
Cerebellum
c-Kit
p-Tyr
tubulin
130
40
C
c-Kit+/+
c-KitTgD814Y c-Kit+/+
ERK2
p-ERK1/2
D
CD45
0.46%
0.42%
E
Wildtype Allele
Targeting Vector
Targeted Allele
PGK-neo
PGK-TK
Probe
c-Kit-/-
F
Liver
Hoechst
E15.5
G
Head
Branchial Arch
Stomach
E11.5

Figure S1

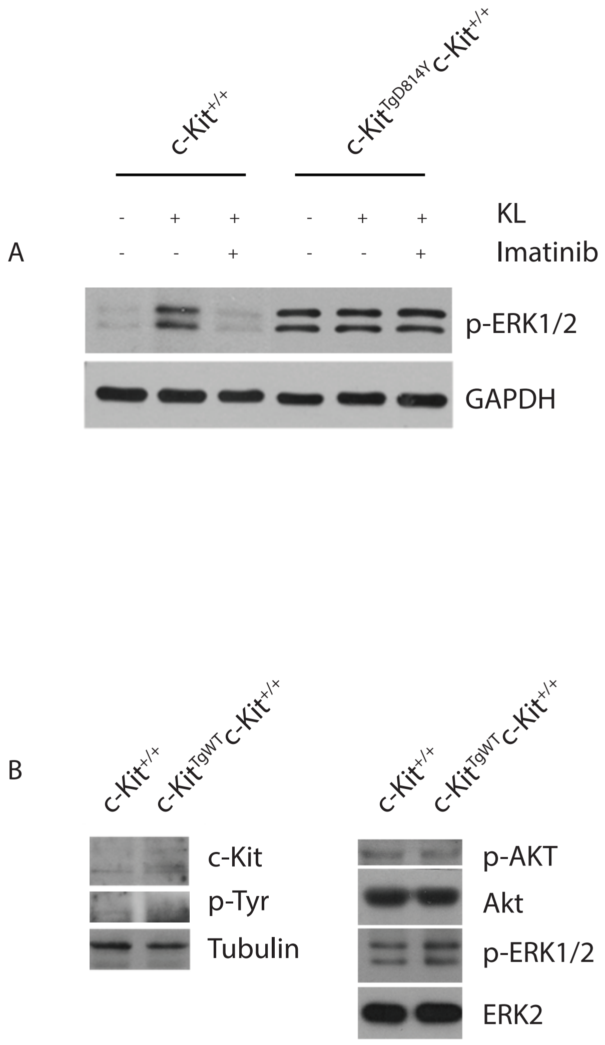
A
c-Kit+/+
c-KitTgD814Y c-Kit+/+
- + + - + + KL
- - + - - + Imatinib
p-ERK1/2
GAPDH
B
c-Kit+/+
c-KitTgWT c-Kit+/+
c-Kit
p-Tyr
Tubulin
p-AKT
Akt
p-ERK1/2
ERK2

Figure S2

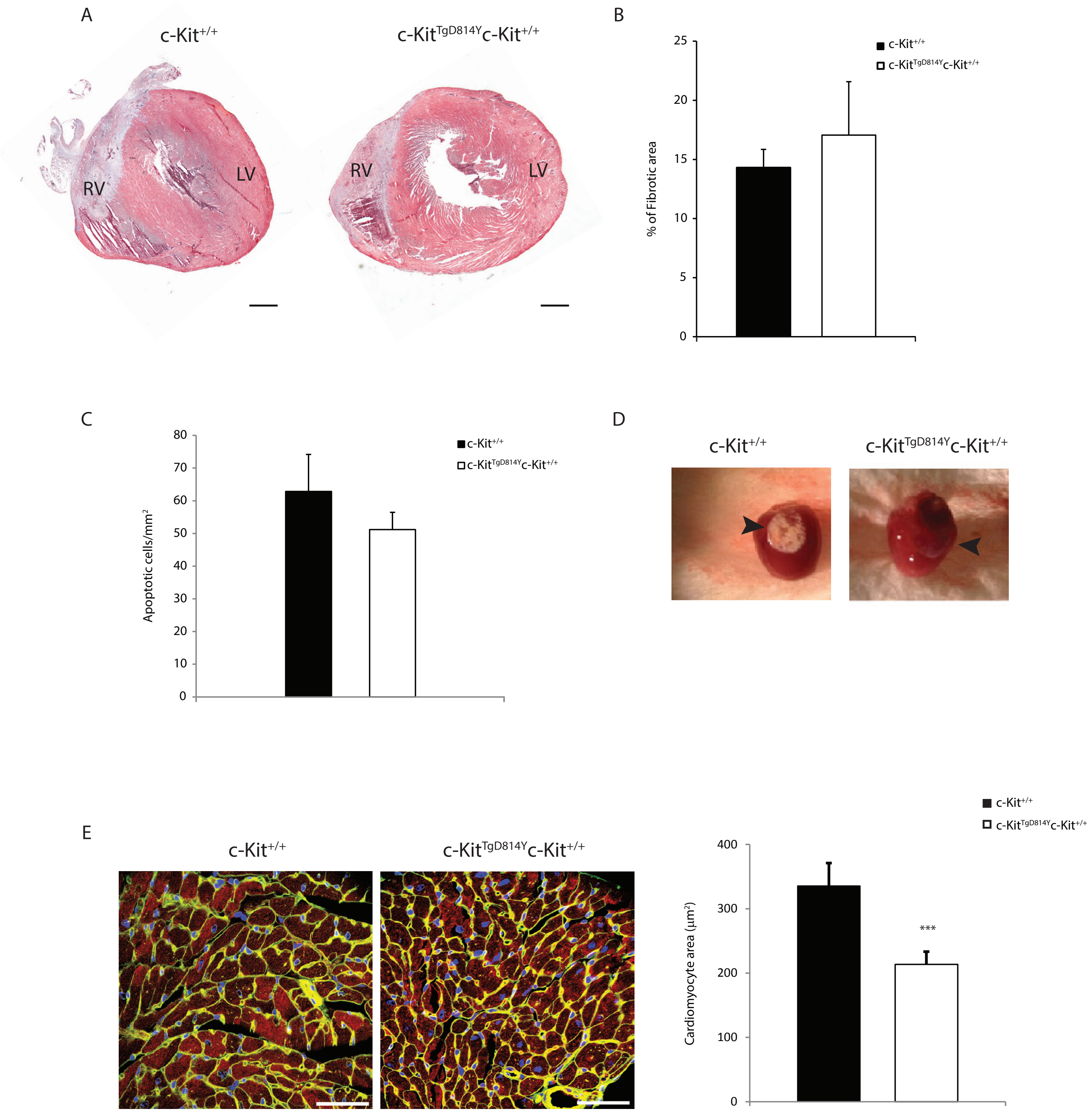
A
c-Kit+/+
c-KitTgD814Yc-Kit+/+
RV
LV
RV
LV
B
c-Kit+/+
c-KitTgD814Yc-Kit+/+
% of Fibrotic area
0
5
10
15
20
25
C
c-Kit+/+
c-KitTgD814Yc-Kit+/+
Apoptotic cells/mm2
0
10
20
30
40
50
60
70
80
D
c-Kit+/+
c-KitTgD814Yc-Kit+/+
E
c-Kit+/+
c-KitTgD814Yc-Kit+/+
c-Kit+/+
c-KitTgD814Yc-Kit+/+
Cardiomyocyte area (µm2)
0
100
200
300
400
***

Figure S3

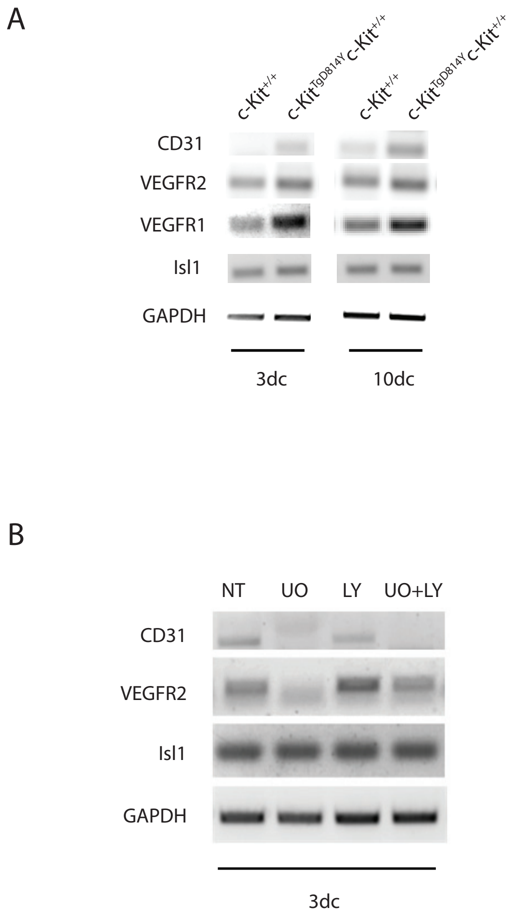
A
c-Kit+/+
c-KitTgD814Y c-Kit+/+
c-Kit+/+
c-KitTgD814Y c-Kit+/+
CD31
VEGFR2
VEGFR1
Isl1
GAPDH
3dc
10dc
B
NT
UO
LY
UO+LY
CD31
VEGFR2
Isl1
GAPDH
3dc

Figure S4

SUPPLEMENTAL FIGURE LEGENDS

Supplementary Figure 1. Expression of c-Kit^{TgD814Y} receptor. A) Increased expression of c-Kit^{TgD814Y} was revealed in 7dpp hearts by immunoprecipitation analysis. B) Increased expression and activation of c-Kit^{TgD814Y} in 7dpp testis and cerebellum was assessed by WB analysis. C) WB analysis in bone marrow extracts from *c-kit*^{TgD814Y}*c-kit*$^{+/+}$ and *c-kit*$^{+/+}$adult mice show similar c-Kit expression and activation. 80 micrograms of proteins were loaded. D) Analysis of hematopoietic stem cell population (CD45^{-}c-Kit^{+}) by flow cytometry in bone marrow confirms no changes in c-Kit^{+} cells percentage (mean intensity values 40.3%±0.82% of *c-kit*^{TgD814Y}*c-kit*$^{+/+}$ vs 41.4%±3.1% of *c-kit*$^{+/+}$) and c-Kit expression due to D814Y mutation. E) Schematic representation for *c-kit*$^{-/-}$ mice generation by inserting a neo cassette in intron 16th and picture of *c-kit*^{TgD814Y}*c-kit*$^{-/-}$ and *c-kit*$^{-/-}$ embryos at 15.5dpc. Lack of rescue of phenotype by the transgene is shown below. Images were taken with a WILD stereoscope coupled to a Nikon Coolpix 4500 camera. F) Immunofluorescence analyses on heart and liver cryosections collected from 15.5dpc *c-kit*$^{+/+}$, *c-kit*^{TgD814Y}*c-kit*$^{-/-}$ and *c-kit*$^{-/-}$ embryos reveal c-Kit staining in *c-kit*^{TgD814Y}*c-kit*$^{-/-}$ compared to *c-kit*$^{-/-}$ hearts. Scale bars 30µm. G) Immunofluorescence analyses of tissue cryosections collected from 11.5dpc *c-kit*$^{+/+}$ and *c-kit*^{TgD814Y}*c-kit*$^{-/-}$ embryos. Scale bars 20µm.

Supplementary Figure 2. A) MAPK phosphorylation was evaluated following 30 minutes of 10µM Imatinib pre-treatment. B) Expression of c-Kit^{TgWT} receptor was revealed in 2 dpp hearts by WB analysis.

Supplementary Figure 3. A) Representative Masson Trichrome–stained myocardial sections from wt and transgenic mice 9 days post-myocardial lesion. Blue, fibrotic tissue and red, viable myocardium. Scale bars 1mm. B) Histogram showing the percentage of fibrotic area in wt and transgenic mice (n=3 mice) 9 days post-myocardial lesion. Areas are measured using Image J. Data are reported ± SE. C) Apoptosis was similar in c-*kit*^{TgD814Y}*c-kit*$^{+/+}$ and *c-kit*$^{+/+}$ mice 9 days post CI. Apoptotic cells were detected by TUNEL assay in the scar and the number of terminal deoxynucleotidyl transferase dUTP nick end labeling stained cells for mm^{2} is reported. D) Representative picture of hearts one month post CI. Arrowheads show scars area in c-*kit*^{TgD814Y}*c-kit*$^{+/+}$ and *c-kit*$^{+/+}$ mice. E) Representative pictures of right ventricles one month post CI. Scale bars 50µm. Histogram shows the percentage of cardiomyocytes area in wt and transgenic mice (n=4 for *c-kit*$^{+/+}$ and n=4 c-*kit*^{TgD814Y}*c-kit*$^{+/+}$ mice) one month post CI. Data are reported ± SD. ***P<0.001.

Supplementary Figure 4 A) Endothelial marker semiquantitative RT-PCR of c-*kit*^{TgD814Y}*c-kit*$^{+/+}$ and *c-kit*$^{+/+}$ CSCs cultured with endothelial cell conditioned medium (dc). B) Representative image of semiquantitative RT-PCR on c-*kit*^{TgD814Y}*c-kit*$^{+/+}$ CSCs for endothelial markers 3 days after incubation with or without ERK1/2 (U0, 10µM) and AKT (LY, 10µM) inhibitors.
